# SCUBA divers as oceanographic samplers: The potential of dive computers to augment aquatic temperature monitoring

**DOI:** 10.1038/srep30164

**Published:** 2016-07-22

**Authors:** Serena Wright, Tom Hull, David B. Sivyer, David Pearce, John K. Pinnegar, Martin D. J. Sayer, Andrew O. M. Mogg, Elaine Azzopardi, Steve Gontarek, Kieran Hyder

**Affiliations:** 1 Centre for Environment, Fisheries & Aquaculture Science, Lowestoft Laboratory, Pakefield Road, Lowestoft, NR33 0HT, UK; 2 School of Environmental Sciences, University of East Anglia, Norwich, NR4 7TJ, UK; 3 Scottish Association for Marine Science, Dunbeg, Oban, Argyll PA37 1QA, UK; 4 NERC National Facility for Scientific Diving, Scottish Association for Marine Science, Dunbeg, Oban, Argyll PA37 1QA, UK.

## Abstract

Monitoring temperature of aquatic waters is of great importance, with modelled, satellite and *in-situ* data providing invaluable insights into long-term environmental change. However, there is often a lack of depth-resolved temperature measurements. Recreational dive computers routinely record temperature and depth, so could provide an alternate and highly novel source of oceanographic information to fill this data gap. In this study, a citizen science approach was used to obtain over 7,000 scuba diver temperature profiles. The accuracy, offset and lag of temperature records was assessed by comparing dive computers with scientific conductivity-temperature-depth instruments and existing surface temperature data. Our results show that, with processing, dive computers can provide a useful and novel tool with which to augment existing monitoring systems all over the globe, but especially in under-sampled or highly changeable coastal environments.

The coastal environment is an important region of our oceans and is vulnerable to pressures brought about by increasing human populations and climate change^1^. The challenges associated with increasing understanding and improving management of this dynamic environment are becoming ever more complex, often requiring large data sets to underpin decision making^2^. Numerous approaches are now in place to bridge this data gap, including satellite remote sensing^3^ and *in-situ* monitoring^4^, but these methods have their limitations (e.g. cloud cover). Another approach that is gaining credibility is citizen science, where volunteers with no formal training collect, categorise, transcribe, or analyse scientific data^5^,^6^. The value of citizen science approaches is recognised by scientists^7^ and decision-makers^8^. One particularly promising new application of citizen science is the use of low cost sensors carried by members of the public on devices like mobile phones and other handheld electronic technology^9^. These can record information at a location and upload the measurements via the internet to a central repository (e.g. noise pollution: http://www.noisetube.net/ and air quality: http://airqualityegg.com/).

Aquatic temperature data are important for understanding how ecosystems function and the long-term consequences of anthropogenic drivers, for example, the steady rise in greenhouse gas emissions, and dynamic changes in temperatures^10^. There is large scale monitoring of sea surface temperature using both remote sensing and *in-situ* platforms^3^,^11^,^12^,^13^, but there is a lack of depth resolved temperature profiles for inshore regions. This is a significant data gap as inshore temperatures are important for commercial activities (e.g. aquaculture and fisheries), development of young commercial fish on nursery ground (e.g. seabass^14^), thresholds for migration and spawning, and long-term trends in the productivity and distribution of species^15^.

‘Animals of opportunity’ have been used as a source of temperature profile data in otherwise inaccessible regions, including seabirds^16^, seals^17^ and human volunteers^18^,^19^. Most divers now carry wrist-mounted ‘dive computers’ that calculate decompression status, but also record profiles of dives in terms of dive time, depth and water temperature^20^. Divers are often keen ‘citizen scientists’ contributing to many scientific programmes^21^ with active social media sites (e.g. http://www.divebuddy.com/) and several social networking platforms to upload dive information (e.g. http://www.movescount.com/). Estimates of the number of active divers globally range from 6 to 10 million^22^. Hence, the scuba diving community represents a huge and novel source of aquatic temperature profile data over large spatial and temporal scales. Compilation of these profiles could augment existing monitoring by enhancing the number of inshore temperature profiles, and provide a resource for scientists to better understand the marine environment and organisms’ responses to changes in the environment.

Despite the potential of scuba diver temperature data to add to existing temperature data sources, no attempt has yet been made to compile and assess the quality of these data. Additionally, often only the minimum temperature of a dive profile is provided, though depth resolved temperature data can be acquired from many dive computer models. To assess the quality of this data, temperature profiles were compared with existing scientifically derived and calibrated data sets of temperature. Diving several models of commercially available dive computers alongside scientific conductivity-temperature-depth (CTD) instruments enabled us to assess the accuracy, precision, time-lag, consistency and sensitivity within and between instruments. Methods were developed to process temperature profiles to enhance the quality of the data. Recommendations of how to use this approach in support of marine science are provided, with results used to assess the potential for temperature profiles from scuba divers to augment existing aquatic temperature monitoring.

## Results

### Collecting dive temperature profiles – the diveintoscience portal

**Figure 1:**
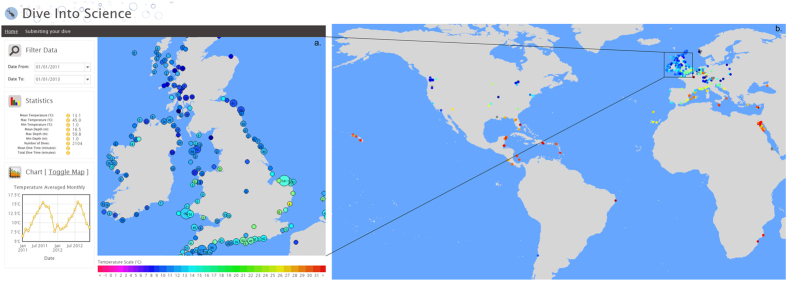
Temperature data from the ‘diveintoscience’ portal. (**a**) Screenshot of the ‘diveintoscience’ interface, where simple query tools and summary data are displayed on the left sidebar calculated for the area displayed on the map. (**b**) Locations of temperature data stored on the “diveintoscience” portal. In map pane a, the circle represents the location of the dive, the size of the circle relates to the number of dives and the colour of the circle represents the average temperature. The screenshot (**a**) was taken from the ‘diveintoscience’ portal (http://www.diveintoscience.org), with both maps (**a**) and (**b**) generated using the ggplot2 (http://CRAN.R-project.org/package=ggplot2) and maps (http://CRAN.R-project.org/package=maps) functions of R (version 3.2.2), which uses publicly available coastline coordinates from the NOAA National Geophysical Data Center (http://www.ngdc.noaa.gov/mgg/shorelines/shorelines.html).

**Figure 2:**
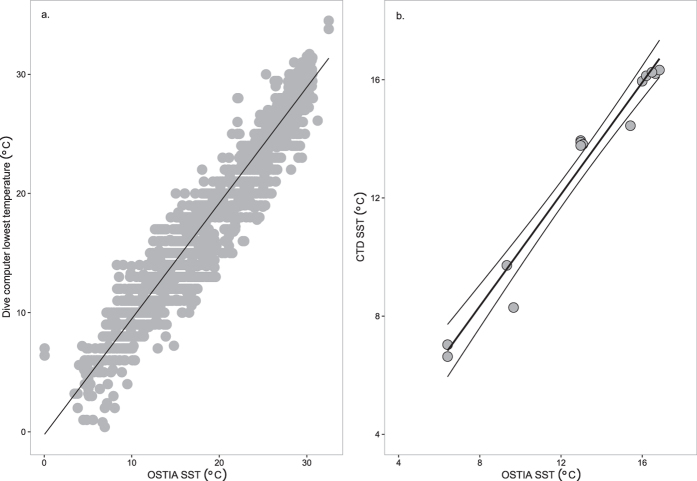
Temperature comparisons. (**a**) Sea Surface Temperature (SST) from the OSTIA database compared to the lowest recorded temperature of the dive computer. (**b**) OSTIA SST compared SST from the surface 10 m of the water-column recorded by the conductivity-temperature-depth (CTD) instruments. The solid lines correspond to the linear fit between the variables, with confidence limits shown in Fig. b.

In total, 7,682 individual dives that had been logged between April 1992 and December 2012 were uploaded to the diveintoscience portal (Fig. 1a,b), corresponding to the minimum temperature of the dive. This demonstrated the ease of collating a large temperature dataset from divers quickly using a citizen science approach. Of the 7,682 dives, 5,078 had corresponding Sea Surface Temperature (SST) values and 225 had corresponding Lake Surface Temperature (LST) values (as extracted from the Operational Sea Surface Temperature and Sea Ice Analysis (OSTIA^3^) database and satellite derived ARC-Lake database^23^, respectively), providing a match for 69% of the data. The temperature difference between surface and lowest temperatures (recorded by the OSTIA SST database and the CTD, respectively), was used to provide an idea of the degree of error between the two temperatures. For sites around the UK, a difference of between −7.1 and 0.7 °C was apparent, with greater differences for deployments in June compared to February or October when the water column was highly stratified (Table 1). The maximum difference between the minimum temperature of the CTD and the OSTIA SST (7 °C) was used to exclude potentially erroneous dive computer data. Of the remaining data, there was a good agreement between the lowest temperature recorded by the dive computers and the OSTIA SST data at the corresponding site and day (r^2^ = 0.95, root mean square error (RMSE) = 1.72, slope = 0.97, intercept = −0.25; Fig. 2a).

### Assessing the accuracy of temperature recorded by dive computers

**Figure 3:**
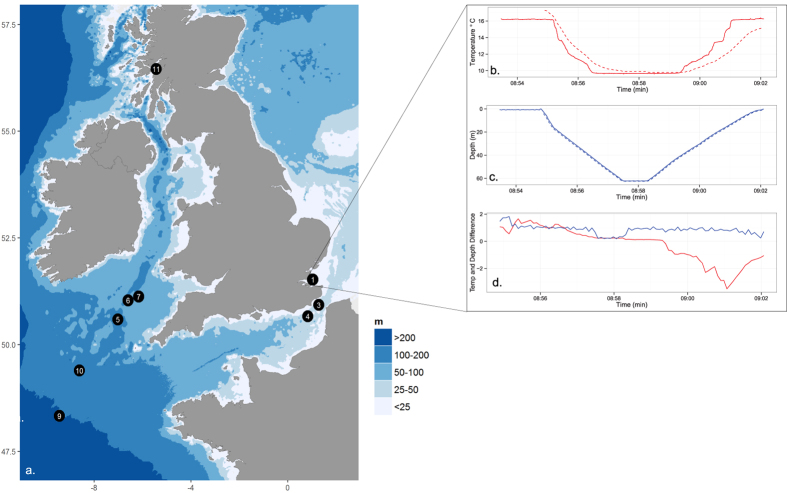
Deployments of dive computers with conductivity-temperature-depth (CTD) instruments. (**a**) Map of CTD and dive computer deployment sites, where site 1 corresponds to deployments 1 and 2, site 7 corresponds to deployments 7 and 8 and site 11 corresponds to deployments 11 to 14. (**b**) Time series of temperature and (**c**) depth recorded by the CTD (solid line) and dive computer model 2 (dashed line) after time stamps were corrected. (**d**) Differences between depth (blue) and temperature (red) recorded by the CTD compared to the dive computer. Map generated using ggplot2 (http://CRAN.R-project.org/package=ggplot2) and maps (http://CRAN.R-project.org/package=maps) functions of R (version 3.2.2).

Understanding the accuracy of temperature recorded by dive computers is essential in order to assess if the data can be used to augment existing aquatic monitoring programmes. As a result, a series of field tests was conducted to assess the accuracy, precision, consistency and sensitivity within and between dive computers in comparison with CTDs. These field tests involved seven dive computer models and two CTD profilers at nine locations on fourteen occasions, between February and October 2014 (Fig. 3a, Table 1).

SST data downloaded from the OSTIA database were compared to the mean temperature within the surface 10 m of the CTD. There was good agreement between these variables (r^2^ = 0.98, RMSE = 0.70, slope = 1.01, intercept = −0.28) with temperature differences ranging from −0.97 to 1.37 °C (Fig. 2b). Raw dive computer data was compared to CTD data deployed at the same time and site. When all 62 dive computer datasets were combined, the dive computer temperature explained 84% of the variance in the temperature data recorded by the CTD, with an average difference between temperature records of 0.02 ± 1.28 °C (Supplementary Table S1). When dive computers were compared to the CTD profiles individually, the average difference was 1.07 ± 0.70 °C (range from −0.34 to 1.38 °C) and the average RMSE was 1.29 ± 0.77 °C (range from 0.36 to 2.63 °C), with variability in both the accuracy and precision of models (Supplementary Table S1).

Within model variation was quantified for dive computer models 1 and 2. Absolute temperature differences at the start of the dive (T_1_) in some cases exceeded 2 °C, though the absolute mean temperature difference over the entire track did not vary by more than 0.7 °C. Increased temperature differences at T_1_ are likely caused by the dive computer heating up prior to deployment, as the air temperature was warmer than the water temperature at the time of deployment.

The minimum temperature recorded by the dive computer was compared to the minimum temperature recorded by the CTD (Supplementary Figure S1a). The minimum temperature recorded by the dive computers explained 95% of the CTD minimum temperatures, and differed by on average 0.15 ± 0.66 °C, with a range of −1.27 to 1.37 °C (RMSE = 0.67). The corresponding depths of these minimum temperatures differed on average by 8.28 m and ranged from 0 to 32.10 m (Supplementary Figure S1b), reflecting temperatures recorded at the same dive depth and recorded at the surface and bottom of the dive, respectively. Disparities between these depths likely relates to the lag in the response of the temperature recording by the dive computers.

### Using dive computer temperature data to augment existing aquatic monitoring

Given the variance in temperature offset reported by several of the dive computers (Supplementary Table S2), the dive computer data was offset-from-zero to attempt to reduce this error. Results from this single point calibration showed 0 °C difference for dive computer model 2, and +1 °C difference for models 1 and 3. Additionally, the temperature lag was estimated for each dive computer using the time required for temperatures to stabilise at a specific depth. For temperatures to reach within 0.5 °C of the CTD temperature at the bottom of the first dive, there was a lag of between 5 and 720 seconds (Supplementary Table S2), with differences between dive computer models and consistencies within models (Supplementary Figure S2). For example, two different dive computers of the same model had similar mean and maximum temperature lag convergence times (Supplementary Table S2). Notably, dive computer model 3 did not reach within 0.5 °C of the CTD temperature at any point during the dive. In situations where complete depth-temperature profiles are available from dive computers, the temperature lag of the dive computer (which can exceed 10 min) can be corrected using the relationship between the rate of change in the temperature and the difference to the “true” temperature as measured by a CTD (Supplementary Table S2). The processing algorithm developed indicated that temperature data from dive computers can be collated and used to augment existing monitoring, and can be sourced from existing historical records or new dives. However, information about the computer model is required to improve the accuracy and utility of the data.

## Discussion

In this study, temperature data collected from the computers of divers has been shown to provide a novel data source. Data uploaded to the ‘diveintoscience’ portal were shown to have reasonable accuracy, demonstrated using comparisons with sea and lake surface temperature data, and through direct comparisons with CTDs and water baths. Most dive computers do not include dedicated temperature sensors but derive their temperature data from the thermal corrections being made to the pressure sensors used to inform the decompression algorithms. The temperature information stored by the computer may be limited by memory capacity which, in turn, influences the recording intervals which affects the quantity and quality of the data. However, simple algorithms can produce robust products that can be used by scientists to assess regional changes in physical oceanography, or to monitor climate change, assess the response of organisms to environmental change, or validate remote sensed data from satellites in coastal regions. Identification of the depth of the thermocline is likely to be possible in the future with a more structured collection of data, but will depend on the strength of the thermocline and the quality of the dive computer. For example, the minimum temperature recorded by dive computers can be compared to surface temperatures at the same location from modelled datasets (like the OSTIA database), providing an indication of coastal stratification. Though, importantly, we may predict a spatio-temporal bias in the regions and profiles sampled, with increased numbers of dives collected in regions with high visibility (stratified water-columns). Overall, our results show that divers can provide an accurate and useful source of aquatic temperature profiles that can augment existing monitoring programmes with global coverage. Though, the use of this data will depend on the scientific question in mind. For example, there may be scenarios where an error of 1 °C is un-acceptable, in which case improvements need to be made in the accuracy and consistency of dive computer temperature records.

The average difference between temperature recorded by the dive computer and by the CTD did not exceed 3 °C, and within model difference was on average less than 1 °C. This is in agreement with results from Azzopardi and Sayer (2012), which tested 47 different dive computer models by 14 manufacturers. The overall outcome indicated that in a range of test temperatures between 10 and 17 °C the median variance was 5.1 °C (with a range of −4.0 to 1.1 °C). In contrast, the temperature difference between surface temperatures recorded by the dive computer and the OSTIA SST database did not differ by more than 1.5 °C. The increased error in temperature readings from the dive computers, and the variability in the accuracy and precision of recorded data is an issue which needs to be resolved if temperature data are to be used to augment current climatologies. For example, the dive computers can have a consistent temperature error (as shown for model 3). By using a zero-offset calibration method, this error can be corrected, providing a simple means to validate temperatures recorded by dive computers. Thus, to improve the validity of uploaded dive computer data, websites focused on dive computer uploads could split uploads into two sections where calibrated and un-calibrated data can be stored separately. Further work is required to understand the effect that the water column structure has on the quality of the data extracted, including the level of variation that it is possible to detect. Additionally, information about the model and brand of the dive computer can be used to assign a level of quality to each upload.

Temperatures at depth are often only available offshore through the analysis of CTD data or modelling^4^,^24^. Many dive computer models store a single temperature value corresponding to the minimum temperature of the dive^20^. In the present study, CTD and dive computer minimum temperatures were relatively similar (within +/−1.5 °C, RMSE = 0.67 °C). When compared to global modelled temperature estimates at depth, RMSE values are higher than in this study (0.73 °C at between 5 and 100 m)^24^, suggesting that this minimum temperature value can be used to provide a reasonable estimate of water temperature with at least comparable accuracy to currently available modelled data. However, the depth that this temperature represents can be anywhere from the water surface to the bottom of the dive. The reason for the disparity between these depths, likely relates to the lag in the response of the temperature sensor of the dive computers, though the maximum depth of the dive can still be used as an indication of the depth of this temperature. Additionally, dive computer manufacturers do not have a standard method of reporting the minimum temperature or the location of this minimum temperature, potentially introducing an additional error. Validation against OSTIA and LST is likely to have removed large geolocation errors, as the temperature variance may exceed the data quality thresholds. An additional cross-validation could be developed to reduce the potential for geolocation errors using the database of dive site names and locations that will be compiled as the ‘diveintoscience’ data set grows. However, geolocation is unlikely to be a problem in the long-term as technology that is not widely available at present, including GPS on dive computes and navigation aids, become more common. Computer manufacturers could then provide a means to store temperature profile information from each dive with georeferenced start and end points, so that users are not reliant on memory to record temperature information or locations.

In situations where dive computers record depth and temperature continuously, more accurate estimates of temperatures at specific depths can be estimated, potentially providing temperatures at multiple depths. Some dive computer models require that divers remain at a constant depth for up to 12 minutes to obtain accurate temperature estimates. However, with further tests, it may also be possible to correct temperature estimates using continuously recorded depth and temperature information for each specific model of dive computer. Estimates of temperatures can be made from the temperature change within a minute at a set depth, rather than waiting for the temperatures to stabilise. Though this method requires further validation, the potential to assign temperatures to specific depths would be of great value.

This study has shown that a large set of depth-resolved temperature measurements can be collected quickly and easily from divers, and provide accurate readings of temperature after correction. It was also possible to get historic temperature information from the logbooks of individual divers meaning that a time series could be produced. As a result, we believe that dive computers are a useful and novel tool which can be used to augment temperature layers at sites all over the globe, but especially in under-sampled or highly changeable coastal environments. The compromise between portability and data quality is important when attempting to use dive computer data to augment existing temperature datasets, with further work required to calibrate and accurately geolocate dives to ensure data quality. Thus, at present, to ensure that dive computer data is accurate, the data needs to be quality assured prior to uploading, to minimise the incidence of errors. A potential method to check the quality of the data at the time of upload would be to record the model and manufacturer of the dive computer at upload, appropriate processing may then be carried out on the uploaded data using algorithms designed for each model type. Additionally, there is potential to work with dive computer manufacturers to improve the quality of the sensors in these devices, allowing divers to directly upload their data without cleaning. This accurate data may then be used firstly, by scientific diving organisations and socials networks to gain a greater understanding of dive sites and secondly, as a source of global sub-surface temperature data to augment or be complimentary to other ocean temperature climatologies (e.g. Argo^11^ or the Hadley Centre sea-Ice and Sea Surface Temperature (HadISST) data set^25^).

In the future, there is great potential to build on the diveintoscience portal to deliver a significant integrated aquatic monitoring interface for divers that goes beyond temperature data. The development of dive computers to include more sensors (e.g. salinity, oxygen) or the deployment of low cost *in-situ* sensors by dive schools and clubs (e.g. wave height) could further add to this potential. Divers generally are keen citizen scientists that are happy to collect information to help better understand the oceans^26^,^27^. Hence, a diver ocean monitoring programme that measures many environmental variables could be developed that delivers high resolution spatiotemporal data, even if only a small proportion of the 6–10 million dives and the many thousands of dive schools and clubs get involved. In addition to depth-temperature profiles uploaded by divers, other sources of temperature information can be collected and integrated into monitoring systems. For example, data collected by other ‘animals of opportunity’ including seals^17^, birds^16^ and fish^28^ may provide (potentially more accurate) *in situ* observations of temperature to fill data gaps in under sampled areas. Though, the quality and location of the temperature recorded will depend on the behaviour of the animal and the tag technology used. Species which frequently sample the entire water column will provide the most useful temperature information, such as, seals^29^ and sea bass^30^. Further exploration of the quality of this temperature and geolocation data are required, with the potential for an integrated monitoring of temperature data (and other variables) recorded using all ‘animals of opportunity’ in combination.

## Methods

### Collecting dive temperature profiles – the diveintoscience portal

A web portal for the collation and visualisation of diver temperature data called ‘diveintoscience’ (http://www.diveintoscience.org) was created using Linux-Apache-MySQL-PHP (LAMP) technology and a post-GIS database (Fig. 1a). Using a citizen science approach, dive profiles were obtained from individual divers, diver social media sites (e.g. Dilogs - http://www.dilogs.com), and other diver-based citizen science projects (e.g. ‘Seasearch’ that maps seabed habitat in the UK - http://www.seasearch.org.uk). A spreadsheet was provided for divers to compile their own computer and logbook data, and email it to the system administrator for quality assurance before uploading into the system. This process was developed instead of a direct upload facility to ensure the quality of the data provided, as initial sets of data acquired had a series of issues including dives in swimming pools. A simple map-based interface was compiled to enable the use of Google Earth to allow users to view information about the temperature at a user-defined selection of sites, including specific summary information, and changes over time (Fig. 1a).

The data comprised over 7,682 readings across the globe (Fig. 1b) and included lowest temperature, maximum depth, latitude, longitude and the date. To get an indication of the degree of error in uploaded dive computer temperature data, uploads were compared with both SST and LST. The OSTIA database provides daily temperature estimates from combined satellite derived and *in-situ* observations of the sea surface temperature at a resolution of 0.05°. The ARC-Lake database provides daily LSTs from a series of (Advanced) Along-Track Scanning Radiometers ((A)ATSRs) at a resolution of 0.05°. Where possible, daily surface temperatures were extracted from these databases for each release site by extracting the corresponding pixel closest to the dive latitude and longitude. Temperature differences between the dive computer temperature and the reported SST/LST were then compared. Disparity between SST/LST and dive computer temperatures can be expected, especially as comparisons are between the lowest temperature recorded by the dive computers and the SST. To account for differences between temperatures, the degree of error between surface and the lowest temperature of dives were derived from OSTIA SST values and CTD lowest temperature values from around the UK (deployments detailed below). This error provided a means to exclude dive computer uploads which were un-realistic. Other potential sources of error within dive computer data are explored using the methods detailed below.

### Assessing the accuracy of temperature recorded by dive computers

CTDs are scientific instruments that are well maintained and frequently calibrated and used to collect temperature profiles. They represent a ‘gold standard’ against which dive computer performance can be accurately judged. For deployments 1 to 10, the CTD system consisted of a CTS-C-1E OEM-type FSI CT sensor (Falmouth Scientific, USA) and a PDR 1828 pressure sensor (Druck, USA) integrated onto an ESM2 profiling logger (Cefas, UK), as used in several other studies^31^,^32^. The FSI temperature sensor was calibrated in-house using a 5010 fluid bath (Guildline, Canada) referenced to two 162CE PRTs (Rosemount, USA) across a F17 resistance bridge (Automatic System Laboratories, UK). Water bath calibrations have shown the FSI CT to be accurate to ±0.005 °C. The dive computers were mounted onto the CTD frame and were deployed to depths of between 13 and 70 m. For deployments 11 to 14, a YSI Castaway portable CTD (SonTek, USA) was used, which is accurate to 0.1 PSU and 0.05 °C with a sampling rate of 5 Hz. The temperature and conductivity sensors are regularly calibrated in-house with a Seabird 19+ and a Seabird 911+ (Seabird, USA) and show good agreement within the manufacturer specifications. Dive profiles for deployments 11 to 13 were selected to maximise the temperature range, and as such were relatively deep (~30 m). The dive profile for deployment 14 was selected to mimic a standard, square profile recreational dive to just over 19 metres (Table 1).

Depths and temperatures were recorded by all dive computers, though sampling rates and resolution varied between models (Supplementary Table S3). Resolutions ranged from 0.1 to 1.0 °C and 0.1 to 1.0 m depth. Sampling frequency varied between 1 Hz and a single temperature per dive, depending on model (either the lowest temperature recorded or temperature at deepest point of the dive).

To enable the comparison of dive computers with CTD data, the time-stamp of each dive computer was aligned to the time-stamp of the CTD using depth as a marker (Fig. 3c). The quality of the dive computer data was assessed by comparing the difference in temperature at the start of the dive (T_1_) with other metrics of temperature error over the entire dive (the average difference and RMSE). Within model comparisons were conducted on models where more than one dive computer was tested at the same deployment site (models 1 and 2), and the precision and accuracy of dive computers were categorised into low or high.

Low and high accuracy were defined as dive computers with a mean temperature difference (compared to the CTD) of less than or more than +/−0.5 °C, respectively. Low and high precision dive computers were defined as dive computers with standard deviation of the temperature differences of less than or more than 0.1 °C, respectively. The minimum temperature and corresponding depth at this minimum temperature were extracted from the CTD and dive computer. Consistencies between the minimum temperature recorded by dive computers compared to a nominal temperature have been explored by Azzopardi and Sayer (2012). However, the depth assigned to this minimum temperature have not been explored and are important when attempting to estimate temperatures at specific depths.

Temperatures recorded by dive computers are not stored in a standard way and vary between models. For example, many brands only provide the minimum temperature of the dive, or the temperature at maximum depth. The minimum temperature and corresponding depth at this minimum temperature were extracted from the CTD and dive computer. Differences between minimum temperatures and depths at minimum temperatures were compared between sites and dive computer models. In addition to comparing CTD data directly with dive computer data, SST data downloaded from the OSTIA database were compared to surface water temperatures from the CTD, providing an indication of the degree of error within the OSTIA SST data. Surface water temperatures from CTDs were calculated as the mean temperature within the surface 10 m.

### Using dive computer temperature data to augment existing aquatic monitoring

To maximise the utility of data produced by dive computers, it is important to develop methods to process the data and return the most accurate and robust output. Linear and non-linear bias in the dive computer temperature may result in significant errors, with potential variability within and between models. In order to reduce this bias, dive computer users can quantify temperature offsets prior to logging their data. However, dive computer users are unlikely to have access to laboratory reference instruments required to quantify temperature bias at multiple temperatures. Thus, a simple single point calibration was tested which is easily implemented by divers outside of the laboratory. The dive computer was immersed in a bucket filled with ice and cold water and stirred vigorously for 10 minutes, after which the dive computer temperature was read and an offset-from-zero determined. Water temperature within the bucket during stirring remained close to 0 °C as assessed by a laboratory PRT (Fisher Scientific Traceable ±0.013 °C at 0 °C).

Algorithms can also be used to obtain more accurate estimates of temperatures at depth. The amount of time required for dive computer temperatures to reach within 0.5 °C of the recorded CTD temperature at a set depth (the bottom of the first dive in this example) was used to estimate the temperature lag. An estimate of the temperature was then made using the scaling relationship between the temperature change within the first minute at the given depth and the temperature difference between the CTD and dive computer (Supplementary Table S2, Supplementary Figure S2). The potential for corrected dive computer data to be used to augment existing aquatic monitoring was then assessed in terms of the new accuracy of the records.

## Additional Information

**How to cite this article**: Wright, S. *et al*. SCUBA divers as oceanographic samplers: The potential of dive computers to augment aquatic temperature monitoring. *Sci. Rep*. **6**, 30164; doi: 10.1038/srep30164 (2016).

## Supplementary Material

Supplementary Information

## Figures and Tables

**Table 1:** The location, depth and summary temperature information for the deployment of dive computers attached to conductivity-temperature-depth (CTD) instruments.

CTD	Site	Date	Location		Dive computer models	Max Depth (m)	Temperature (°C)				OSTIA SST (°C)	SST – OSTIA (°C)	Min – OSTIA (°C)
			Lat.	Long.			Min	Max	Range	SST			
Cefas ESM2 CTD	1	03/02/2014	51.53	1.04	4	15.62	6.91	7.43	0.52	7.03	6.41	−0.62	0.50
	2	03/02/2014	51.52	1.02	4	14.05	6.43	7.25	0.82	6.63	6.41	−0.22	0.02
	3	03/02/2014	50.93	1.28	4	13.67	9.63	9.84	0.21	9.72	9.33	−0.39	0.30
	4	03/02/2014	50.67	0.83	4	21.29	8.16	8.42	0.26	8.29	9.66	1.37	−1.50
	5	16/06/2014	50.59	−7.03	1, 2, 3	51.91	9.68	16.08	6.4	15.95	16	0.05	−6.32
	6	17/06/2014	51.04	−6.60	1, 2, 3	51.51	9.41	16.05	6.64	16.13	16.2	0.07	−6.79
	7	18/06/2014	51.13	−6.16	1, 2, 3	62.32	9.59	16.4	6.81	16.21	16.65	0.44	−7.06
	8	19/06/2014	51.13	−6.16	1, 2, 3	69.33	9.90	16.87	6.97	16.33	16.85	0.52	−6.95
	9	21/06/2014	48.33	−9.44	1, 2, 3	62.40	13.49	14.56	1.07	14.45	15.42	0.97	−1.93
	10	22/06/2014	49.39	−8.61	1, 2, 3	61.66	9.85	16.4	6.55	16.25	16.48	0.23	−6.63
YSI Castaway portable CTD	11	09/10/2014	56.46	−5.44	6, 7	28.20	13.55	17.33	3.78	13.94	12.97	−0.97	0.58
	12	10/10/2014	56.46	−5.44	6, 7	30.31	13.61	16.36	2.75	13.88	12.97	−0.91	0.64
	13	14/10/2014	56.46	−5.44	6, 7	31.33	12.29	13.91	1.62	13.81	13.08	−0.73	−0.79
	14	16/10/2014	56.46	−5.43	6, 7	19.15	12.83	16.43	3.6	13.77	12.97	−0.80	−0.14
