# Supplementary material for: SCUBA divers as oceanographic samplers: The potential of dive computers to augment aquatic temperature monitoring
Source: Sci Rep. 2016 Jul 22;6:30164. doi: 10.1038/srep30164 (PMC4957074; doi:10.1038/srep30164)
Supplement: Supplementary Information [file srep30164-s1.docx]

**SCUBA divers as oceanographic samplers: The potential of dive computers to augment aquatic temperature monitoring**

Serena Wright^1*^, Tom Hull^1^, Dave Sivyer^1^, David Pearce^1^, John K. Pinnegar^1,2^, Martin D.J. Sayer^3,4^, Andrew O.M. Mogg^3,4^, Elaine Azzopardi^3,4^, Steve Gontarek^3^, and Kieran Hyder^1^

^1^ *Centre for Environment, Fisheries & Aquaculture Science, Lowestoft Laboratory, Pakefield Road, Lowestoft, NR330HT, UK.*

^2^ *School of Environmental Sciences, University of East Anglia, Norwich, NR4 7TJ, UK*

*^3^ Scottish Association for Marine Science, Dunbeg, Oban, Argyll PA37 1QA, UK.*

^4^ *NERC National Facility for Scientific Diving, Scottish Association for Marine Science, Dunbeg, Oban, Argyll PA37 1QA*

**Corresponding author*

**Supplementary information**


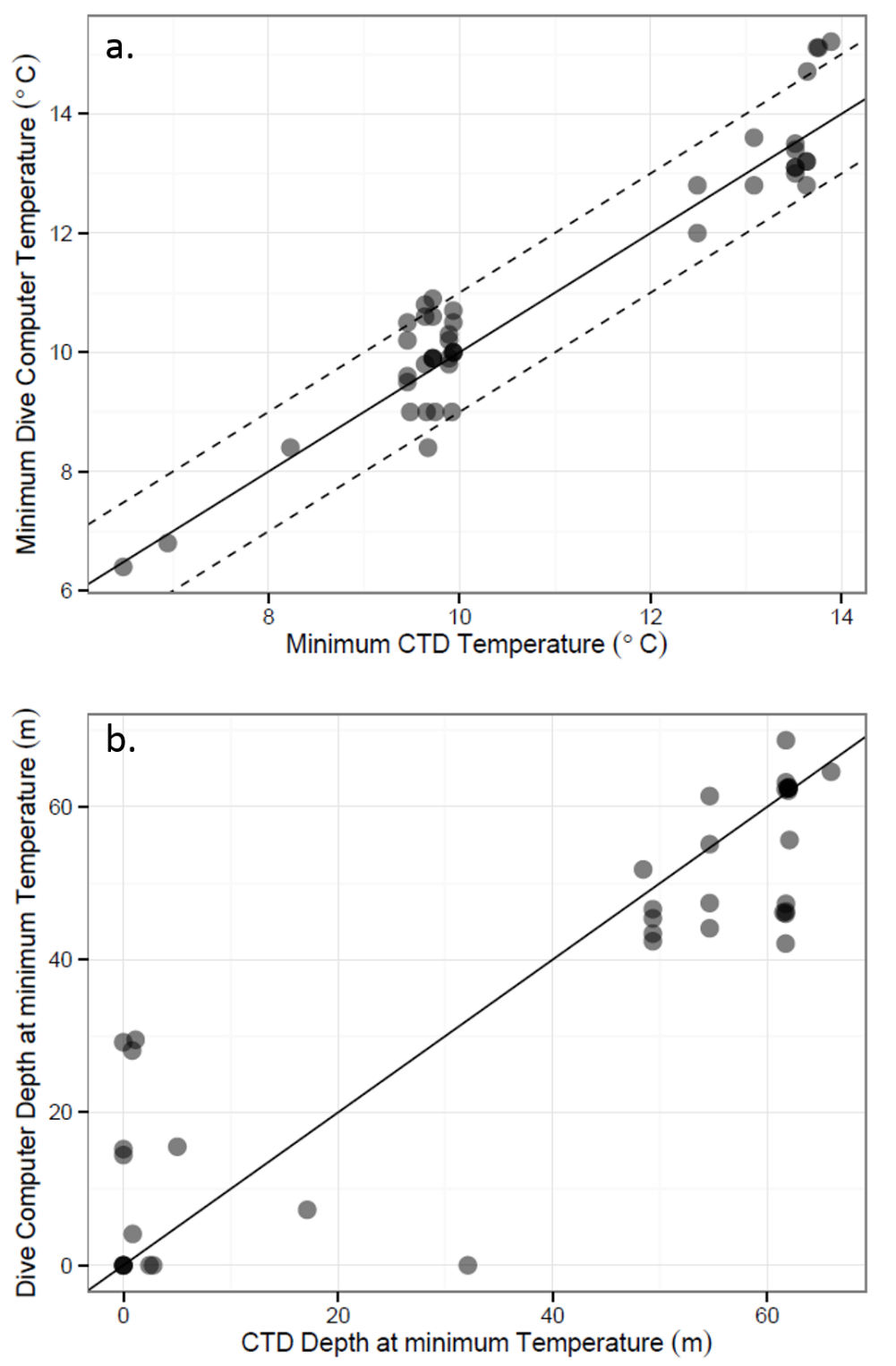


Supplementary Figure S1. (a) CTD minimum temperature compared to dive computer minimum temperature, where the solid line corresponds to a one to one fit with +/- 1°C. (b) Corresponding depth at this temperature for the CTD and dive computer.


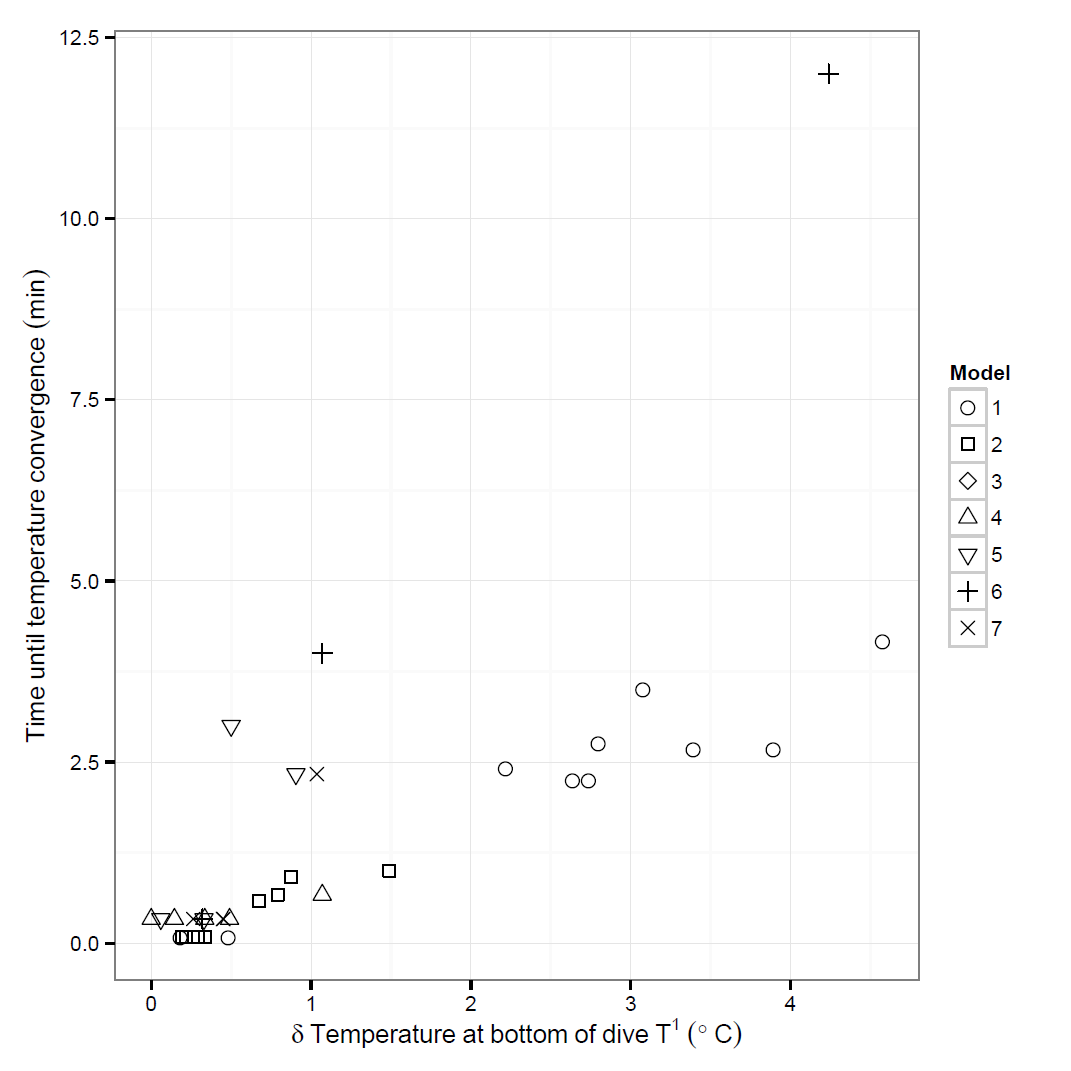


Supplementary Figure S2. Temperature difference at the bottom of the first dive compared to the time required for temperatures to converge to within 0.5 °C of the CTD temperature.

Supplementary Table S1. Temperature difference (average and root mean squared error, RMSE) between dive computers and CTDs. N corresponds to the number of dives analysed for each dive computer. Precision and accuracy are defined as low (L) or high (H).

| Model | ID | N | Mean temperature difference (°C) | Mean RMS (°C) | Max RMS (°C) | Precision | Accuracy |
| --- | --- | --- | --- | --- | --- | --- | --- |
| 1 | a | 6 | 0.72 ± 0.31 | 2.63 ± 0.56 | 3.20 | L | L |
|  | b | 6 | 0.35 ± 0.35 | 1.79 ± 0.62 | 2.65 | L | H |
| 2 | a | 6 | -0.04 ± 0.17 | 1.05 ± 0.37 | 1.46 | L | H |
|  | b | 5 | -0.23 ± 0.07 | 1.07 ± 0.28 | 1.46 | H | H |
| 3 | a | 4 | 1.38 ± 0.08 | 1.40 ± 0.09 | 1.54 | H | L |
| 4 | a | 6 | -0.34 ± 0.27 | 1.28 ± 0.45 | 1.89 | L | H |
| 5 | a | 4 | 0.13 ± 0.34 | 0.43 ± 0.15 | 0.60 | L | H |
| 6 | a | 3 | -0.18 ± 0.38 | 0.36 ± 0.05 | 0.41 | L | H |
| 7 | a | 4 | -0.10 ± 0.05 | 1.40 ± 0.09 | 1.54 | H | H |
|  | Total | 44 | 0.18 ± 0.56 | 1.29 ± 0.77 | 1.57 ± 0.92 |  |  |

Supplementary Table S2. Time required for temperatures recorded by dive computers to reach within 0.5 °C of the CTD temperature and the correlation between temperature change (TC) within 1 minute at a set depth compared to the temperature recorded by the CTD (TT)

| Tag Model | ID | Mean temperature difference (°C) | Time for temperatures to converge (s) | | |  | TT = a + TC * b | | | | |
| --- | --- | --- | --- | --- | --- | --- | --- | --- | --- | --- | --- |
|  |  |  | Min | Max | Mean |  | a | | b | | P |
| 1 | a | 3.01 ± 1.57 | 5 | 250 | 150 |  | 0.254 | | 2.301 | | <0.0001 |
|  | b | 2.18 ± 1.16 | 5 | 210 | 141 |  |  |  |  |  |  |
| 2 | a | 0.44 ± 0.27 | 5 | 85 | 29 |  | -0.107 | | 1.500 | | 0.0036 |
|  | b | 0.72 ± 0.59 | 5 | 85 | 42 |  |  |  |  |  |  |
| 3 | a | 1.76 ± 0.93 |  |  |  |  |  | |  | | 0.1565 |
| 4 | a | 0.41 ± 0.41 | 20 | 100 | 37 |  | 0.030 | | 1.776 | | 0.0804 |
| 5 | a | 0.45 ± 0.35 | 20 | 180 | 90 |  |  | |  | | 0.1300 |
| 6 | a | 1.88 ± 2.08 | 20 | 720 | 327 |  | -0.249 | | 1.642 | | 0.0029 |
| 7 | a | 0.55 ± 0.34 | 20 | 140 |  |  |  |  | |  | |

Supplementary Table S3. Temperature and depth resolution, accuracy and sampling frequency for diver’ decompression computers

| Brand | Model | Resolution | | Accuracy | | Sampling frequency (s) |
| --- | --- | --- | --- | --- | --- | --- |
|  |  | Temp (°C) | Depth (m) | Temp (± °C) | Depth (m) |  |
| X | 1 | 0.10 | 0.10 | 2.00 | 0.20 | 20 |
|  | 2 | 0.10 | 0.10 | 2.00 | 0.20 | 20 |
|  | 3 | 0.05 | 0.10 | 2.00 | 0.20 | 5 |
| Y | 4 | 1.00 | 1.00 | 2.00 | 1.00 | 5 |
| Z | 5 | 0.40 | 0.02 | - | - | 4 |
|  | 6 | 0.40 | 0.10 | - | 0.20 | 4 |
|  | 7 | 0.40 | 0.10 | - | 0.20 | 4 |

Unreported values are shown as (-).
